# Genetic Engineering and Encapsulation Strategies for *Lacticaseibacillus rhamnosus* Enhanced Functionalities and Delivery: Recent Advances and Future Approaches

**DOI:** 10.3390/foods15010123

**Published:** 2026-01-01

**Authors:** Leontina Grigore-Gurgu, Florentina Ionela Leuștean-Bucur, Gabriela-Elena Bahrim

**Affiliations:** Faculty of Food Science and Engineering, “Dunărea de Jos” University of Galati, 800008 Galați, Romania; leontina.gurgu@ugal.ro (L.G.-G.); florentina.bucur@ugal.ro (F.I.L.-B.)

**Keywords:** *L. rhamnosus*, CRISPR-Cas9, functional foods, gut–organ axis, encapsulation systems

## Abstract

This review addresses the recent advances made through various genetic engineering techniques to improve the properties of *Lacticaseibacillus rhamnosus*, not only for industrial applications, but also for the health-related benefits. However, due to the strict regulations on microorganisms intended for human consumption, concerning the insufficient characterization degree of the newly isolated strains and the lack of data regarding the safety of the genetically modified (GM) variants, the feasibility of bringing such *L. rhamnosus* strains to the market and their safety prospects were evaluated. Given their multiple in vivo functions in the contexts of synbiotic and symbiotic functionality, *L. rhamnosus* strains are more than classic probiotics and need furthermore attention. In the functional food context, this review highlights the impact of *L. rhamnosus* derived bioactives on the human gut–organ axis, pointing out recently demonstrated molecular mechanisms of action with the host’s gut microbiome to reduce the negative effects of obesity and its related metabolic disorders, as well as depression and Parkinson’s disease, as the major challenges confronting humans today. Beyond that, considering *L. rhamnosus* delivery and its postbiotics accessibility to consumers via functional foods, notable progress was made to enhance their stability by developing various encapsulation systems, which are also emphasized.

## 1. Introduction

Researchers from all over the world together with food industry experts show an increased interest in developing functional foods enriched with metabiotics (pre-, pro-, post-, and paraprobiotics) [1]. This trend derives from the growing recognition of the health-related benefits of probiotics and derivates, particularly in preventive health and wellness. In general, probiotics are exposed to multiple stress factors during gastrointestinal transit, which can compromise the cells’ functional efficacy. Thus, a critical point in probiotic formulation to benefit from its effectiveness is to maintain viable cells count higher than 10^6^ CFU/g after ingestion [2]. In this context, *Lacticaseibacillus rhamnosus* (*Lactobacillus rhamnosus*) is one of the most studied probiotic species within the *Lacticaseibacillus* genus regarding fermentation processes carried out to obtain bioactive compounds with enhanced functionality. Several strains of this lactic acid bacterium (LAB) meet the necessary criteria to be qualified as probiotics, as they are able to withstand the stress conditions associated with the human gastrointestinal tract, to adhere to the gut’s epithelial cells and colonize it, and to prevent the pathogenic microorganisms’ proliferation due to their biofilm forming ability [3]. However, the functional benefits attributed to *L. rhamnosus*, as a probiotic species, are correlated with its proven biological activities within the host. Due to the recognized its benefits, it is estimated that *L. rhamnosus* market value (USD 1.2 billion in 2024) will double by the year 2033 [4].

In this regard, in the food engineering sector, different encapsulation techniques have been applied to *L. rhamnosus* to maintain a high viability in the human intestine, ensure its participation to the metabolic and immune processes and facilitate health benefits through microbiota–gut–brain axis. The choice of a suitable encapsulation method from a wide range of technologies (such as spray drying, freeze-drying, emulsification, coacervation, liposomal delivery, electrospraying) depends on several critical factors related to the size of capsule, the biocompatibility of encapsulating materials with the probiotic strain, and the intended food, pharmaceutical, or nutraceutical applications [5].

Commonly, the *L. rhamnosus* exceptional adaptability and persistence in the food matrix or human gastrointestinal tract are effects of its pan-genome structure expression. Adaptation to these conditions is possible due to the expression of different essential genes responsible for carbon sources utilization, cell envelope-related functions, or antimicrobial activities [6].

In order to explore the genomic and phenotypic diversity within the *L. rhamnosus* species, Douillard et al. [6] isolated 100 strains from various ecological niches, including human body sites (77 strains) and dairy products (23 strains). The whole-genome sequencing showed that gene content among the isolated strains varied significantly (with a median of 96.7%), when reads were aligned to the *L. rhamnosus* GG (*L*GG) reference strain. More precisely, the authors emphasized that genes content similarities or divergences relative to *L*GG expressed adaptation to specific ecological niches [6]. Furthermore, even though the complete set of *L*GG genes (3016) were reported in eleven strains of human origin and four strains isolated from dairy products, some particularities among strains regarding carbohydrate metabolism were observed. More precisely, the genes encoding phosphatase transfer systems (PTS) or various carbohydrates transporters, play a key role in strains adaptation to specific niche [6]. In a recent study, Huang et al. [7] explored the core and accessory gene content of 70 *L. rhamnosus* strains, isolated from infant and adult Chinese subjects. The main findings regarding carbohydrate utilization are related to the presence of genes encoding glycoside hydrolases (GHs), offering an integrative image about genetic diversity within species, and the possibility of using them as probiotic bioingredient [7].

This review aims to provide a comprehensive synthesis of the current knowledge and developments regarding the beneficial potential of *L. rhamnosus* strains, with a particular focus on their probiotic characteristics, which were enhanced by genetic engineering techniques, or preserved by encapsulation methods for both food industry applications and host functionality.

## 2. Improvement of *L. rhamnosus* Strains Through Genetic Engineering for Industrial Applications

**Figure 1:**
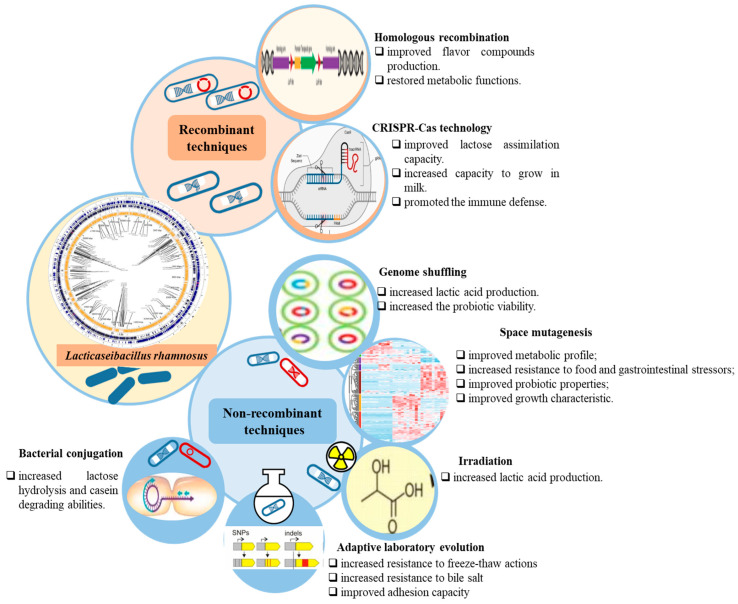
Genetic engineering techniques to enhance *L. rhamnosus* metabolic functionalities along with their associated achievements.

Genetic engineering used to explore LAB’s functionalities, such as substrate metabolism, metabiotics’ production, and tolerance to stress, represents an ongoing research effort. To enhance *L. rhamnosus* beneficial characteristics, a variety of genome engineering tools have been employed (Figure 1). These techniques were both recombinant and non-recombinant. The recombinant techniques, such as homologous recombination, and CRISPR-Cas technology, can result in either genetically modified organisms (GMO), which contain DNA fragments originating from different species, or self-cloning organisms (SCO), which do not contain heterologous DNA or do not produce proteins other than those occurring naturally. The non-recombinant methods generate strains that are not considered as GMO by the legislation currently in force. These can, in turn, be untargeted non-recombinant strategies, including random mutagenesis and adaptive laboratory evolution (ALE), and targeted recombinant strategies, as is the case with bacterial conjugation [8].

### 2.1. Recombinant Strategies

#### 2.1.1. Homologous Recombination Based on Exogenous Vectors

Homologous recombination based on exogenous vectors has been used to both delete and integrate genes of interest in the LAB’s genomes. Briefly, the strategy relies on an integrative plasmid that carries a construct containing two homologous regions through which the recombination process takes place and antibiotic resistance genes used as selection markers. The plasmid integrates into the host’s genome following a single crossover event via one of the homologous regions, being then eliminated after the second one. Depending on the region where the second crossover event occurred, either wild-type (the same homologous region) or mutant (the other homologous region) cells can be obtained [8]. This genome integration strategy allows permanent interventions, meaning that the recombinant genes remain stable without a selective pressure [9]. Plasmid-based homologous recombination allowed researchers to establish the role of some genes in *L. rhamnosus* probiotic traits. For instance, construction and analysis of a *luxS* knockout *L*GG mutant and its genetic complementation strain led to the conclusion that *luxS* gene plays a central role in the activated methyl cycle and sulfur amino acid metabolism, which are essential for growth and biofilm formation, rather than being involved in quorum sensing through production of AI-2 signaling molecules [10].

Besides assessing the specificity of gene knockout experiments, gene complementation can also be used as a strategy to restore some metabolic functions in *L. rhamnosus* strains. One *L. rhamnosus* strain unable to produce diacetyl and acetoin while growing in milk, as a consequence of a frameshift mutation in the *als* gene encoding for acetolactate synthase, became functional by transferring a wild-type *als* gene from a strain yielding high amounts of this flavor compound. The restored *L. rhamnosus* defective strain behaved similarly to the strains naturally producing elevated amounts of diacetyl and acetoin during cheese ripening [11].

#### 2.1.2. CRISPR-Cas Technology

Clustered Regularly Interspaced Short Palindromic Repeats—associated protein (CRISPR-Cas) systems are adaptive immune mechanisms against mobile genetic elements encountered in prokaryotic organisms. These systems are used to intercept and destroy the invasive nucleic acids injected by bacteriophages or brought along with exogenous plasmids based on the memory of prior infections provided by the short DNA fragments (spacers) integrated in the CRISPR array, whose transcripts (single pre-CRISPR RNAs—crRNAs) guide the Cas nucleases to the specific cleavage sites (complementary protospacers) [12,13]. There are two classes of CRISPR-Cas systems, which are in turn divided into several types. Class 1 systems include type I, III, and IV systems together with their subtypes and class 2 systems comprise type II, V, and VI systems and their respective subtypes [14]. Class 1 systems use a multiple-protein effector complex such as Cascade (CRISPR-associated complex for antiviral defense), which is specific to the type 1 systems, to recognize and degrade the foreign DNA elements [15], while class 2 systems rely on single large proteins such as Cas9 to perform the effector function [16]. The valid CRISPR-Cas loci identified in *L. rhamnosus* strains have been reported to belong to the subtype II-A system (Lsal1 family) which encompasses Cas1, Cas2, Cas9, and Csn2 proteins [6,17]. This makes *L. rhamnosus* a suitable probiotic bacterium for industrial fermentation applications, as the high prevalence of the subtype II-A CRISPR-Cas systems confers great stability and efficiency in cases of phage contaminations [17]. The same is suggested by Pujato et al. [18] who conducted a comparative analysis of 8 strains of *L. rhamnosus* with other *Lacticaseibacillus* species regarding their spacers homology to foreign genetic elements and concluded that the subtype II-A system in this bacterium is more active in acquiring spacers following phage infections [18].

Moreover, it was shown that, similarly to *Streptococcus pyogenes*, *L. rhamnosus* possesses an RNA-based regulatory structure located upstream of the CRISPR array that promotes the immune defense against the most recent invaders. This works by crRNAs production from the newest spacers acquired. The underlying mechanism consists of a stable stem-loop structure formation between the pre-crRNA (precursor CRISPR RNA) leader and the first repeat bordering the newest spacer that enables tracrRNA (trans-activating CRISPR RNA) hybridization with the second repeat and speeds up crRNA processing by RNase III so that it can bind to the Cas9 nuclease [19].

The *L*GG derived Cas9 protein, termed LrCas9, was reported to be more efficient and specific in genome editing than LbCas12a, SpCas9-NG, or SpRY due to its preference for targeting A/T-rich PAM sequences (5′-NGAAA-3′). LrCas9 has been successfully used in the development of genetic engineering tools, such as vectors designed for targeting multiple genes simultaneously, CRISPRi (CRISPR interference) for genes repression, and CRISPRa (CRISPR activation) for genes overexpression [20]. More precisely, the authors proved that the LrCas9 enzyme maintains genome-editing activity even at lower temperatures, enabling precise genetic modifications in key crops such as rice, wheat, tomato, and larch [20]. By supporting the development of higher-yielding, and more nutritious crops, LrCas9 could accelerate innovation across the food supply chain, ensuring more sustainable food products. The high abundance of type II CRISPR-Cas systems in species belonging to the *Lacticaseibacillus* genus offers as well great potential for precise engineering of their genomes by repurposing them [12]. However, applying CRISPR-Cas technology for genome editing in these species might be challenging for several reasons, such as the following: limited homologous recombination ability which makes it difficult to edit multiple genes simultaneously, high cell death rate as a consequence of double-strand breaks, insufficient expression of sgRNA resulting in gene targeting failure, off-target effects leading to modification of non-specific DNA sequences, low rates of the systems delivery through cells transformation, and limited availability of PAM (Protospacer Adjacent Motif) sequences in the strains’ genomes [21]. In spite of these, Xie et al. [22] managed to correct the mutations at the level of the *lacTEGF* operon which were responsible for *L*GG inability to assimilate lactose by successfully reprogramming the bacterium’s native type II-A CRISPR-Cas9 system. The authors’ strategy consisted of constructing an editing plasmid containing the sgRNA placed under the control of a strong constitutive promotor and a repair template including the two homologous regions flanking the target sites. The spacers sequences of 30 bp length were selected so that the target sites were located upstream the PAM sequence recognized by the Cas9 enzyme and linked to the tracrRNA via an artificial loop to form a unique sgRNA molecule. Compared to the parental strain, the transformed cells were capable of inducing milk coagulation during incubation at 37 °C by decreasing the pH as a result of lactic acid production. Also, unlike LGG cells, the modified ones increased significantly (~1.21 log_10_ CFU/g growth) during yogurt fermentation, maintaining their viability at a higher rate under storage at low temperatures as well [22].

This important achievement gives rise to new research directions in the field of probiotics as CRISPR-Cas tools enable to engineer strains which not only exhibit greater stability, but also enhance the functional food properties, including shelf life, fermentation efficiency, or nutrients bioavailability.

### 2.2. Non-Recombinant Strategies

#### 2.2.1. Random Mutagenesis as Untargeted Non-Recombinant Strategy

*Genome shuffling* is an evolutionary engineering method applied to increase the metabolites production of industrial microbial strains by improving their ability to metabolize specific substrates or withstand specific stressors present in the growth media [23]. Genome shuffling occurs through the non-homologous recombination of the multi-parental strains’ genomes with relevant phenotypes induced via random mutagenesis and selection [24]. This technique is often used together with protoplasts generation to increase the chances of the recombination process [25]. Genome shuffling strategy was applied to enhance *L. rhamnosus* ATCC 11443 strain tolerance towards glucose and acidity in order to increase its capacity to produce L-lactic acid. Both objectives were achieved by generating populations with the desirable properties via ultraviolet irradiation and nitrosoguanidine mutagenesis and recursive protoplast fusion application for several rounds. The improved *L. rhamnosus* strains were able to tolerate glucose concentrations of up to 200 g/L and pH levels of up to 3.8, demonstrating a significantly higher capacity regarding L-lactic acid production compared to the wild-type variant [26].

All these advancements offer significant advantages for food industry, particularly in the food fermentation industry. The new trait of *L. rhamnosus*, namely a more efficient glucose metabolism which increases lactic acid synthesis, enables rapid acidification which supports food safety and natural preservation. Moreover, it may contribute to a consistent development of the desirable sensory characteristics, improve the texture, or ensure the probiotic viability without compromising the product quality.

*Space mutagenesis* involves the exposure of organisms to the physical and chemical conditions of space, such as microgravity, high radiation, high vacuum, and extreme temperatures [27]. The changes in the organisms’ genetics, physiology, and metabolism, as a result of the stress induced by these extreme conditions, have the potential of generating new varieties with better traits [28]. However, the altered behavior of the evolved microbes must be comprehensively evaluated for potential harmful effects, like increased resistance to antibiotics, transfer of virulence factors between microorganisms, enhanced biofilm forming ability, or secondary metabolites production [29]. Several studies have reported that exposure of *L. rhamnosus* Probio-M9 strain to space conditions improved its growth characteristics, metabolism, probiotic properties and resistance towards stress factors that can be encountered in food matrices or in human gastrointestinal tract. The mutant cell line R7970 was able to form larger colonies on MRS agar compared to the Probio-M9 cells, this phenotype being associated with an increased capsular polysaccharides (CPSs) production related to the discovered mutation in the *ywqD* gene. The isolate had a better metabolic profile, particularly in glucose utilization, which led to higher lactic acid production, tryptophan metabolism, and bioactive compounds production, such as nicotinic and picolinic acids. The abundance of CPSs was also considered the reason for the mutant’s increased resistance to acidic, osmotic, heat, bile salt, and oxidative and fermentation stress [30]. Regarding their probiotic potential, CPSs not only provide an increased resistance to beneficial bacteria along the human gastrointestinal tract but also exert nutraceutical and bioactive effects [31]. Other *L. rhamnosus* Probio-M9 space mutants (HA-R7970-29, HG-R7970-18, HG-R7970-29), selected based on their superior growth characteristics, were evaluated regarding some probiotic properties, such as antibacterial activity against common pathogenic bacteria, including *Escherichia coli*, *Salmonella enterica*, *Listeria monocytogenes*, *Staphylococcus aureus*, *Bacillus cereus*, and *Pseudomonas aeruginosa*, auto-aggregation ability, cell surface hydrophobicity, and antioxidant activity [32]. Except for the HG-R7970-29 isolate, which, compared to the other strains, exhibited a stronger inhibition against *P. aeruginosa*, the mutants’ antibacterial activity against the six pathogens was comparable to that of the parental strain.

The auto-aggregation ability was significantly higher in Probio-M9 cells, while, regarding the hydrophobicity of the strains, only the HA-R7970-29 isolate reached a value similar to that of the wild-type strain [32]. The advantages regarding the mutants’ antioxidant capacity, in terms of 2,2-diphenyl-1-picrylhydrazyl (DDPH) free radicals, hydroxyl radicals, superoxide anions scavenging ability, differed based on the tested media (bacterial suspensions or fermentation supernatants) and the strain. Overall, the fermentation supernatants outperformed the cellular suspensions, likely due to the secreted metabolites. HG-R7970-18 strain showed the best DDPH and hydroxyl radicals scavenging ability, while the best superoxide anions scavenging ability was registered in the case of HA-R7970-29 strain [32].

Finally, the comparison between *L. rhamnosus* Probio-M9 strain and another isolate obtained following space mutagenesis, HG-R7970-41, concerning the metabolism of 190 single carbon sources, revealed a difference in the carbon metabolism pattern between these strains. The HG-R7970-41 mutant strain exhibited a significantly lower metabolic activity for certain carbon sources, including D-glucose, D-salicin, D-galactose, D-trehalose, N-acetyl-D-glucosamine, D-sorbitol, D-tagatose, D-fructose, D-mannose, and D-melezitose, which was linked to the changes in the phosphorylation and glycolysis pathways as a result of the related genes’ downregulation. From the industrial point of view, such a strain might contribute to the reduction of the costs associated with the substrate acquisition, given that it requires less energy to grow [33].

*Irradiation* is an uncontrolled genetic alteration method that has allowed the selection of lactic acid bacteria strains with improved traits to be used in the fermentative industry [34]. Lin [35] subjected *L. rhamnosus* 6013 strain to microwave radiation and the resulting mutated strains were screened for increased lactic acid production capacity. By applying a microwave radiation frequency of 2450 MHz for 3 min, it generated a mutant strain which, in the presence of glucose as the single carbon source, showed a 1.5-fold increased production of the metabolite compared to that of the wild-type strain. The amplified fragment length polymorphism analysis performed on the mutant *L. rhamnosus* 6013 cells indicated as possible causes for their enhanced lactic acid production the mutations occurred at the malate/lactate dehydrogenase and pyruvate kinase genes level [35].

*Transposon mutagenesis* uses mobile genetic elements, known as transposons or “jumping genes”, which induce random chromosomal mutations by shifting from one position in the genome to another [36]. The specialty literature concerning *L. rhamnosus* genetic modification by transposon mutagenesis is still scarce. Biswas et al. [37] attempted to create a *L. rhamnosus* LRB mutants’ library by using the ISS1 transposon delivered via the pGhos9::ISS1 thermosensitive plasmid, which would have allowed them to identify the genes involved in the bacterium’s response to acid stress. The ISS1 transposon failed to integrate into the *L. rhamnosus* LRB genome, the possible reasons of this advanced by the researchers being the presence of a high number of native insertion sequences (IS) and IS-like elements or host encoded factors like nucleoid associated proteins. Overall, it was concluded that ISS1 transposon is not an appropriate tool for genome modification in this species [37].

#### 2.2.2. Bacterial Conjugation as Targeted Non-Recombinant Technique

Horizontal gene transfer through bacterial conjugation is a naturally occurring process that requires the presence of self-transmissible plasmids or integrated conjugative elements containing the genes involved in the DNA transmission [38]. The DNA transfer between the donor and the recipient cells takes place through specialized structures encoded by the *tra* operon of the conjugative plasmid that span the cell envelope [39]. *Lactococcus lactis* and *Lactococcus lactis cremoris* are common carriers of conjugative plasmids that encode useful traits in dairy fermentation, such as lactose, citrate, and casein metabolism, resistance to bacteriophages and heavy metals, and bacteriocin production [39].

Through bacterial conjugation, a new *L*GG strain was developed with lactose metabolism and proteolytic activity, enabling efficient growth in milk and generating a non-GMO strain suitable as starter culture for dairy industry applications [40]. More precisely, the lactose hydrolysis and casein degrading abilities were achieved by co-culturing the *L*GG with *L. lactis* subsp. *cremoris* NCDO 712, which carries the pLP712 plasmid containing the *lacABCDFEGX* operon responsible for the lactose metabolism and utilization and *prtP* gene encoding for a serine protease [40].

### 2.3. Adaptive Laboratory Evolution

ALE is an experimental technique used in the field of biotechnology for the purpose of generating evolved microbial strains with the desired traits by exposing a population to certain environmental conditions or selection pressures over the course of many generations. This method allows obtaining information regarding the molecular mechanisms’ evolution and the corresponding adaptive phenotypes that occur in the studied strains [41]. Experimental evolution under stress factors that mimic the conditions encountered in the human gastrointestinal tract may help understand how *L. rhamnosus* evolves genetically and how these changes impact its beneficial properties. Freezing and thawing stress may affect the viability of the starter cultures used in dairy industry [42]. A group of researchers attempted to obtain a stable *L*GG variant by employing the ALE technique. Subjecting this strain to freeze–thaw–growth for 150 cycles resulted in mutants with higher survival rate (up to 86.3%) compared to the parental strain, after 6 days of frozen storage in 0.3 M trehalose. Further, the evolved *L*GG mutants displayed faster exponential growth and a shorter lag phase, with a mean doubling time of 13 min shorter and a 29 min reduction in lag phase compared to their ancestor strain [43]. Genome sequencing of the evolved cells revealed genetic mutations primarily altering the cell wall and membrane physical properties [43]. Specifically, the improved resistance to freeze–thaw stress, and, consequently, the increased survival rate, was attributed to functional defects in the *dacA*, *murQ*, *cls*, and *wze* encoding genes. These alterations led to an incomplete cell wall or membrane structure, resulting in a more flexible cell envelope that enables cells to better withstand the mechanical forces generated by ice crystal formation compared to the parental strain [43].

Another study revealed that subjecting *L. rhamnosus* to bile salt stress for approximately 750 generations led to the activation of IS elements, resulting in the deletion of a large chromosol region located on the same genomic island, which includes the *spaCBA-srtC1* pilus gene cluster, in all the adapted cells. Although the loss of piliation occurred to a lesser extent under shear stress, this also highlights a potential risk of mechanical stress on the strain’s genomic stability during industrial processing and handling [44].

However, evolution of bacteria under in vivo conditions such as superior organisms’ digestive tract represents a more complex approach that may result in strains with improved probiotic properties for dietary applications. This is the case of the study recently conducted by Mark et al. [45], whose purpose was to generate *L*GG variants with increased resistance to bile salt stress and improved adhesion to epithelial cells by repetitively administering the probiotic bacteria to mice lacking a functional immune system. Compared to the wild-type cells, the evolved isolates demonstrated a better retention in the mice gut and higher growth rates in the presence of bile salt, which were associated with the identified changes in the genes encoding for cell surface proteins and sortase enzymes, and ATP binding proteins and proton pumps, respectively. Surprisingly, the Caco-2 cell adhesion assay showed a slightly reduced binding capacity of the evolved variants compared to that of the wild-type strain, a fact that could be attributed to some putative mutations in the pili-related genes, as previously noticed by other studies [44].

### 2.4. Legislative Aspects on a Global Level

Genetic engineering of microorganisms intended for human applications is subjected to rules that may differ from one country to another. For instance, due to the difference in the risk perception, the European Union (E.U.) regulations on GMOs use in food and feed production [46] address the authorization procedure and its subsequent steps in a more comprehensive and rigorous manner compared to those in the United States [46,47]. While, in the process of the risk assessment, for precautionary reasons, the E.U. authorities consider GMO-based food and feed products fundamentally different from the conventional versions, in the U.S., these are treated as equivalents to the homologous non-GMO products as long as the scientific evidence does not indicate any possible hazard. Other major differences between the laws of these territories are related to the labelling of GMO-based food and feed products and their traceability, which, in the case of U.S. producers, are not mandatory [48]. However, an exception to the non-mandatory labelling of GMO-based products in the U.S. refers to the possible allergens that do not occur naturally in the non-GMO products [49]. Authorization of GMOs and GMO-based products for production and commercialization presents a different approach according to the country. While at the E.U. level, the decision is up to the political bodies, in the U.S., the process is depoliticized, with the state not participating directly [48]. In the EU, commercialization of GMOs as such or feed and food products containing GMOs falls under the jurisdiction of the Directive 2001/18/EC, which establishes the rules regarding deliberate GMOs release in the E.U. environment. According to the Regulation (EC) No. 1829/2003, before reaching the market, the applicants have to go through a multi-step procedure which starts with the notification of the national authorities and the subsequent request transmission to the European Food Safety Authority (EFSA) Based on the EFSA scientific opinion formulated following a rigorous evaluation of the GMOs risk to human and animal health or the environment from the genetic alteration process perspective, the Commission drafts a decision on the request approval or rejection. Then, the draft decision is subjected to the vote of the Standing Committee on Plants, Animals, Food, and Feed (PAFF Committee), which is formed of the Member State representatives. The PAFF Committee has to approve or reject the authorization by qualified majority. If the qualified majority is not reached, the Commission can summon an Appeal Committee or take responsibility for the decision [50]. In the US, the GMOs approval for placing on the market is up to three federal agencies, namely US Food and Drug Administration (F.D.A.), US Environmental Protection Agency (EPA), and US Department of Agriculture (USDA), which work together to ensure their safety for human, animal, and plant health and to monitor their impact on the environment. In this case, the main goal is to check whether the GMO-based products are safe according to their intended use [51]. In addition, in comparison to the E.U., the U.S. still lack a legal framework regulating GMOs, specifically GM probiotics, as a distinct product category, a situation that is encountered as well in other large countries, such as China [52]. These discrepancies can negatively affect the international trade of such GMO-based products and the advancement of genetic engineering for sustainable food production and food security ensuring [53].

To date, the acceptable genome engineering techniques for LAB modification in the E.U. are those which can occur naturally, such as bacterial conjugation, phage transduction, natural competence, and the random mutagenesis induced chemically or via irradiation. At the same time, the methods that are not currently approved are those aiming at chromosome modification or foreign DNA expression by the use of exogenous vectors, recombineering, and CRISPR-Cas derived systems [54]. Consumer perceptions of GMOs are multilateral, with factors such as cultural, social, and economic contexts, as well as awareness and trust in the regulatory bodies, playing a decisive role in their attitude towards GM food [55]. The degree of consumers’ acceptance with respect to foods derived from GMOs in the U.S. seems to surpass that of those in the E.U. [54].

### 2.5. Evaluation of L. rhamnosus and GM Probiotics Safety

Before obtaining probiotics with additional or improved functions for the benefit of human health, it is necessary that the parental strains be genetically modified and carefully evaluated in terms of safety and resilience [53]. *L. rhamnosus* strains isolated from different sources, including different geographical origins or food matrices, can present a high variability regarding the genetic characteristics [56]. Hence, sequencing the probiotic strains’ complete genomes to be precisely identified and classified, as well as reviewed for any genetic trait of concern, becomes mandatory to ensure their safety [57]. Also, clinical trials evaluating the efficacy and long-term effects of probiotic candidates need to be carried out under a standardized legal framework [58].

Some probiotics can naturally contain mobile genetic elements (MGEs) carrying antibiotic resistance genes (ARGs) [59]. Therefore, there is a high concern that the regular intake of probiotics for health benefits could lead to the dissemination of ARGs among the normal intestinal microbiota and occasionally occurring opportunistic species, rendering the future antibiotic therapies less effective [60]. The AR traits in *L. rhamnosus* strains were generally found to be of low frequency [59]. However, any microorganism intended for human consumption needs to be investigated for the possibility of ARGs transfer to other organisms, if present, and virulence factors [61]. It was shown that the vancomycin resistance factor of the frequently consumed probiotic *L*GG strain is not related to the *van* genes of the opportunistic pathogenic species *Enterococcus faecalis* and *Enterococcus faecium*, being stated that this AR trait is not a transferable one [62]. The erythromycin resistance in *L. rhamnosus* Oxy and Pen probiotic strains is determined by the *erm* chromosomal genes which, in the absence of carrying plasmids or other MGEs, are also less capable of horizontal transfer [63]. In addition to this, the decrease in the susceptibility of *L*GG to erythromycin and ciprofloxacin after repetitive exposure to these macrolides is more likely to be generated by phenotypical alterations or point mutations rather than the associated resistance mechanisms, meaning that the acquired resistance has a low potential of being transferable [64]. The failure in putting into evidence by molecular methods the AR genetic determinants in some *L. rhamnosus* strains identified as being resistant to either clindamycin, gentamicin, or tetracycline above the cut-off concentrations established by EFSA also indicates that these genes may be encoded at the chromosomal level [65]. Overall, according to the studies mentioned above, there is a low probability that AR traits from *L. rhamnosus* strains will be transmitted to other microorganisms mostly because of their intrinsic nature.

Toxigenicity, pathogenicity, allergenicity, immunological impact, and genetic stability over time are other important aspects to be considered in the case of probiotic candidates for human use. The toxicity studies performed on the potential probiotic strain *L. rhamnosus MP108*, including genetic and oral toxicity assays, did not put into evidence any safety issue [66]. However, although *L. rhamnosus* belongs to the normal human flora and is generally considered innocuous, this species can generate infections in certain circumstances. One study reported *L. rhamnosus* as the etiological agent of nosocomial bloodstream infections and, by corroboration of the results with other cases presented in the literature, it was concluded that, in the cases of hospitalized patients, the main associated risk factors were immunosuppression and catheters placement [67]. Other predisposing conditions leading to *L. rhamnosus* infections in humans could be gastrointestinal tract disruption, malignancy, complex surgeries or prior exposure to antibiotics [68]. On the other hand, many studies have reported significant benefits of diets supplemented with *L. rhamnosus* on food allergies and allergic diseases thorough modulation of the host intestinal microbiota and immune response [69,70,71]. Probiotics’ immunomodulatory activity is still questionable. Some strains were shown to exert positive effects, while others were shown to trigger a pro-inflammatory response [72]. The related studies conducted on *L. rhamnosus* generally indicated promising immunomodulatory potential of this bacterium, with limited side effects. However, the possibility of an exaggerated immune response or immune suppression in certain contexts needs to be further investigated [73,74].

The research area concerning the health impact of genetically modified (GM) probiotics in vivo is still narrow, with quite limited trials on animals and no clinical trials to date [52]. Although not including GM *L. rhamnosus* strains, there are a few studies conducted on animal models which demonstrated that some GM-LAB strains could be safely integrated into the food chain. The mice fed with LABs containing plasmids for nutraceuticals overproduction showed a similar behavior in terms of growth rate, food and water intake, hematological parameters, and organs’ relative weight, morphology, and histology, to that of those fed with the progenitor strain [75]. Another study analyzed the interaction between nattokinase producing GM-LABs and rats’ intestinal microflora, also evaluating organs, such as liver, spleen, and kidney, and blood for bacteria translocation. After feeding the rats for 4 weeks with both wild-type and GM strains, the concentration of the beneficial *Bifidobacterium* spp. in the rats’ feces increased significantly compared to that in the control group. At the same time, the harmful bacterium *Clostridium perfringens* remained undetectable in all the groups throughout the experiment. Neither non-GM-LAB nor GM-LAB strains negatively affected the rats’ overall health, and no bacterial translocation occurred [76].

On the other hand, there are scientists who claim that GM probiotics should be prohibited for therapeutic purposes because of their unpredictable behavior in relation to the resident bacteria within the human gut and host’s immune system [77]. Besides the intended effects, genetic modification in LABs may lead to unexpected side effects on consumers [34].

Among these, potential risks including the transfer of ARGs, multidrug resistance genes, and other MGEs to the gut microbiota, may compromise the host’s microbial resistance profile. Additionally, GM probiotics may produce harmful proteins or enzymes that trigger inflammation and disrupt gut microbiome balance, potentially inducing chronic disease [78].

Another main concerns regarding the safety of GM probiotics are their uncontrolled spreading and the transmission of the novel genetic traits to other organisms if accidentally released in the environment [79]. As universally applicable rules to avoid these events, it must be ensured that no antibiotic resistance gene is included in the GM bacteria’s genomes and that the exogenous DNA template used in the engineering process comes from GRAS (Generally Recognized as Safe) microorganisms, such as food grade vectors derived from LAB or bifidobacteria cryptic plasmids [80].

## 3. *L. rhamnosus* as an Active Health Promoter Included in Food Production

### 3.1. Beneficial Effects of L. rhamnosus Metabiotics

Metabiotics involve bioactive and functional complexes of the viable probiotics and inactivated intact or ruptured cells (paraprobiotics), their metabolites and signaling molecules which have been shown to specifically improve the host physiological functions and regulate the interconnection between host metabolic and behavioral functions and gut microbiome diversity, composition, and functionality [81]. The concept of metabiotics emerged following the need for a safer approach in biotherapy using live probiotics, due to their better documented composition and function. Some other advantages offered by metabiotics are as follows: extended shelf-life of food products, lack of side effects, and positive effects on human health [82]. Some representative beneficial postbiotics are short chain fatty acids (SCFAs), antimicrobial compounds, functional peptides, bacteriocins, vitamins, peptidoglycans, lipoteichoic acids, polysaccharides, or polar lipids [81,82]. Paraprobiotics are obtained from probiotics subjected to the inactivation process. Cell disruption can be achieved through different methods, which can be classified into thermal treatments [pasteurization (<100 °C), sterilization (≥100 °C)] and non-thermal treatments (ionizing radiation, ultraviolet rays, high pressure, sonication, dehydration, and pH modification). Other technological strategies are emerging methods, such as pulsed electric field technology, ohmic technology, and supercritical fluids–CO_2_ technology. When choosing an inactivation method, it is important to consider preserving or improving as well as possible the beneficial properties [83]. *L. rhamnosus* derived metabiotics showed various beneficial effects for human health. The cell wall (CW1505) and peptidoglycan (PG1505) of *L. rhamnosus* CRL1505 strain exhibited important immunoregulatory effects on co-cultured human intestinal epithelial cells and dendritic cells when challenged with TLR4 (toll-like receptor 4). MCW1505 had a stronger stimulatory effect, increasing the production of TNF-α, IL-1β, IL-6, and IL-8, while PG1505 was more efficient in improving the production of IL-10, which is closer to the effect of live and UV inactivated *L. rhamnosus* CRL1505 cells [84]. Metabiotic extract from the cell free supernatant obtained following the culture of *L. rhamnosus* MD 14, a strain isolated from infant feces, which consisted primarily of carboxylic acids, ethers, amides, and esters, showed high cytotoxicity against Caco-2 (86.8%) and HT-29 (86.3%) cells. It also slowed down the proliferation of the colon cancer cells by blocking the cell cycle in the G0/G1 phase [85]. Moreover, the metabiotic extract, later shown to contain SCFAs, such as butyrate, acetate, and propionate, acetamide, thiocyanic acid, and oxalic acid, showed promising results as antitumorigenic agent when administered in Sprague-Dawley rats with induced colon carcinogenesis. Administered daily in a medium dose (2 mL/kg) for 6 weeks, the metabiotic extract determined the reduction of fecal pro-carcinogenic enzymes, oxidants, and aberrant crypt foci, downregulation of oncogenes, such as K-ras, β-catenin, Cox-2, nuclear factor kappa B (NF-κB), and upregulation of tumor suppressor p53 gene [86].

The freeze-dried postbiotic extract obtained from *L. rhamnosus* LRH-B2 cells, containing bioactive compounds such as flavonoids, SCFAs, antioxidant enzymes, and organic acids, was shown to inhibit more than 50% of the populations of pathogenic bacteria *L. monocytogenes* ATCC 13932, *E. coli* ATCC 25922, *S. enterica* serovar Typhimurium ATCC 14028, and *S. aureus* ATCC 64542 after their co-incubation at 37 °C for 24 h. The extract exerted low cytotoxicity in MRC 5 and IPEC-J2 cell lines (10% at highest concentration tested of 150 mg/mL) and was not hemolytic to human erythrocyte cells [87].

### 3.2. Postbiotics and Their Functional Effects on Obesity and Related Metabolic Disorders

**Figure 2:**
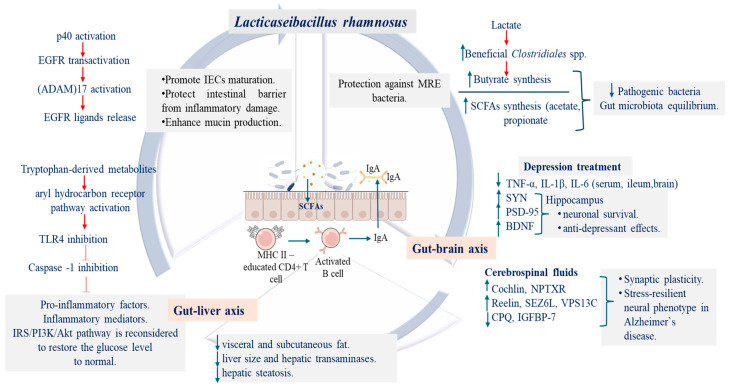
Schematic representation of *L. rhamnosus* in vivo effects in protecting intestinal barrier and modulating the gut–liver and gut-brain axes. SCFAs-short chain fatty acids; MHC II-Major Histocompatibility Complex class II; IgA-immunoglobulin A; EGFR-Epidermal Growth Factor Receptor; (ADAM)17-A Disintegrin and Metalloproteinase 17; TLR4-toll-like receptor 4; NLRP3-Nucleotide-binding domain, Leucine-rich family, Pyrin domain containing 3; IRS/PI3K/Akt-Insulin receptor substrate/Phosphoinositide 3-kinase/Protein kinase B; TNF-tumor necrosis factor; SYN-synaptophysin PSD95-postsynaptic density protein-95 BDNF-brain-derived neurotrophic factor; NPTXR-Neuronal pentraxin receptor; SEZ6L-Seizure 6-Like Protein; VPS13C-Vacuolar Protein Sorting 13 Homolog C; CPQ-carboxypeptidase Q; IGFBP-7-Insulin-like Growth Factor-Binding Protein 7. The blue upward arrow symbol emphasizes increased synthesis or expression, while the downward decreased synthesis or expression.

Obesity is a multifactorial disease characterized by hypertrophy and hyperplasia of adipocyte, being induced by consumption of high-fat diets (HFD), which are responsible for systemic metabolic disturbance, including insulin resistance, lipid metabolism imbalance, and gut microbiota dysbiosis. Once dysbiosis was initiated, the proteins from epithelial tight junction are degraded and the lipopolysaccharides (LPS) are translocated into liver via portal circulation, disrupting the gut–liver axis homeostasis and further activating inflammatory markers. To counteract these effects, Sun et al. [88] used the postbiotic compounds derived from *L. rhamnosus* HF01 strain (cell-free supernatant, CFS) to treat the obesity symptoms developed by mice fed for 12 weeks with HFD. Their results emphasized that, after CFS administration, beneficial bacteria able to synthesize SCFAs (acetic acid and propionate) or those having antagonistic activity against pathogenic bacteria were increased, leading to an equilibrium on gut microbiota diversity. Furthermore, the aryl hydrocarbon receptor pathway was activated through important synthesized metabolites, such as glutaric acid, tryptamine, indole-3-acetic acid, and 5-hydroxyindoleacetic acid, resulting in the formation of some specific compounds having the role to suppress the pro-inflammatory factors, inflammatory mediators, quorum sensing of pathogenic Proteobacteria, and to improve insulin sensitivity, restoring the gut–liver axis activity [88,89] (Table 1, Figure 2).

In a meta-analysis study conducted by Nazarinejad et al. [90], it was highlighted that postbiotics (such as SCFAs, or heat-kill probiotics) derived from different other probiotics significantly reduced the body weight and fat, ameliorating the negative effects developed once obesity has set in, through some default mechanisms. In a similar way, *L*GG administrated (as probiotic or heat-inactivated) to rats fed with high-fat and high-fructose diets proved its efficacy in obesity management, decreasing the visceral and subcutaneous fat, the liver size and hepatic transaminases, while also preventing hepatic steatosis and attenuating the metabolic markers linked to the dysregulated glucose metabolism [91] (Table 1). Regarding the action mechanisms involved in obesity intervention, it seems that postbiotics administration induces downregulation of genes from adipogenesis and lipogenesis process along with an increase in expression of markers linked to lipolysis and β-oxidation. Additionally, it was observed that genes encoding key enzymes from de novo synthesis of fatty acid synthase (FAS) and acetyl-CoA carboxylase (ACC), have a decreased expression [92]. Even though, in the light of the recent findings, the therapeutic potential of *L. rhamnosus* derived postbiotics in managing metabolic disorders is well emphasized, future research is needed to identify which is the exact bacterial compound involved in the beneficial observed outcomes.

### 3.3. L. rhamnosus and Its Psychobiotic Potential

Major depressive disorder (MDD) is a mental health condition characterized by prolonged periods of sadness, dissenters, or lack of pleasure in daily activities; it is also closely related to suicide, particularly among young people. Unlike temporary emotional fluctuation, depression can deeply impact personal, social, and professional aspects of life. These conditions are more prevalent in woman than in man, with global estimates that 3.8% of the global population is affected (4% of men, 6% of women, 5.7% of older adults over the age of 60) [93,94]. Despite medication availability, there is no defined protocol for a definitive cure of depression. Also, for low- and middle-income countries, access to treatment is limited due to insufficient healthcare resources or persistent social stigma associated with mental disorders [93]. Recent studies highlighted a significant connection between gut microbiota and depression, actively mediated by the gut–brain axis. More precisely, individuals with depression exhibit distinctive alterations in gut microbiota composition, having elevated levels of certain microorganisms included into the families of Actinomycetaceae, Enterococcaceae, Leuconostocaceae, Porphyromonadaceae, and Streptococcaceae [66,95] and a higher abundance of *Oscillibacter* and *Parabacteroides* genera [96]. In the study of Bai et al. [95], the relation between gut microbiota and MDD has been explored using both liquid chromatography–mass spectrometry (LC-MS) for serum metabolites spectrum and 16S rRNA gene sequencing for gut microbial composition, on a total of 60 individuals diagnosed with MDD. The results were compared with those obtained from the health control group (HCs). The analysis revealed that of the 24 metabolites identified in the MDD group, 10 of them were linked to inflammatory processes being also involved in three inflammation-associated metabolic pathways. Further, gut microbiota analysis identified 17 genera with differential abundance, predominantly from the Firmicutes phylum, influencing four inflammation-related pathways. There were also highlighted five metabolites (LysoPC(16:0), deoxycholic acid, docosahexaenoic acid, taurocholic acid, and LysoPC(20:0)) as related inflammation biomarkers, showing a strong correlation with the Firmicutes phyla and having a high accuracy in distinguishing MDD from HCs individuals [95].

**Figure 3:**
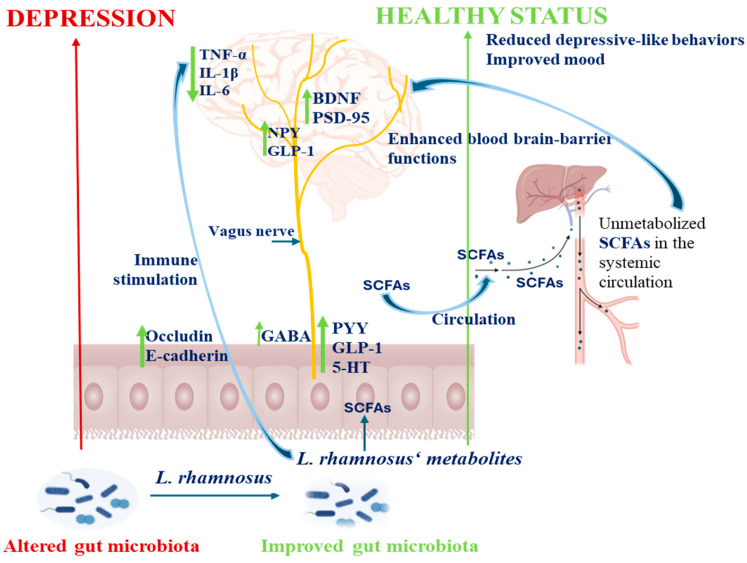
Schematic representation of different molecular mechanisms (neural, endocrine, immune, and metabolic pathways) highlighting antidepressant effects of *L. rhamnosus* and its metabolites (SCFAs) by gut–brain axis modulation. SCFAs stimulate enteroendocrine cells to release GLP-1 and PYY, which act through vagal nerve signaling and modulate neurotransmitter systems, especially GABA serotonin pathways, leading to increase BDNF expression. SCFAs may cross the blood–brain barrier and enhance neuronal function, reduce neuroinflammation (by downregulation of TNF-α, IL-1β, IL-6) in specific brain regions, and increase the level of PSD-95, alleviating depressive behaviors, respectively. The green upward arrow symbol emphasizes increased synthesis or expression, while the downward decreased synthesis or expression.

Since 2005, the use of probiotics as a complementary strategy in treating depression has gained interest due to their favorable profile, low incidence of side effects, or high tolerability [97]. From the *Lactobacillus* genus, *L. rhamnosus* has been the most extensively studied probiotic demonstrating in vivo effective actions in reducing depression symptoms (Table 1). For instance, Pan et al. [98] observed that after 3 consecutive weeks of LGG and antibiotics administration to mice chronically exposed to ethanol (CEE), the gut *Lactobacillus* spp. levels was restored, the interleukins and the tumor necrosis factor (TNF)-α concentrations decreased in the serum, ileum and brain, while the expression of the synaptophysin (SYN) and postsynaptic density protein-95 (PSD-95) together with the brain-derived neurotrophic factor (BDNF) increased in the hippocampus. All of these indicate protective or recovery effects of neural function [98] (Figure 3).

The BDNF is an important neurotrophin family member having important roles in supporting neuronal growth, to maintain their structure and function, being expressed at high levels in hippocampus, cerebral cortex, and basal forebrain, areas which are vital for learning, memory, and thinking. In vivo studies with rats chronically subjected to stress, a significant decrease in hippocampal BDNF level was observed while following treatment with *L*GG, the BDNF levels were found to increase considerably [69,99] (Table 1, Figure 3). Similar results regarding upregulation of BDNF expression were obtained in depressive-like rodent models with the *L. rhamnosus* CCFM1228 strain [100]. Enhanced levels of brain BDNF contribute to neuronal survival and may enhance the upregulation of neurotransmitter receptors while promoting gamma aminobutyric acid (GABA) synthesis (Figure 3). This neurotrophic activity is likely to influence the regulation of receptors for serotonin, dopamine, and norepinephrine, which plays crucial roles in mitigating the symptoms of depression. Thus, the administration of *L*GG demonstrated antidepressant effects, and in some cases showed greater efficacy than conventional antidepressants, such as bupropion and venlafaxine [99].

In another study, Aktas et al. [101] examined the effects of *L. rhamnosus* E9 strain administration on dopaminergic markers and oxidative stress in 1-methyl-4-phenyl-1,2,3,6-tetrahydropyridine (MPTP)-induced mouse model of Parkinson’s disease (PD) (Table 1). It has been shown that after MPTP administration, the expression of dopamine receptor D1 (DR1) level significantly increased while the level of DR2 was unchanged.

**Table 1:** Experimental in vivo framework detailing information of *L. rhamnosus* strains administration for therapeutic effects.

Experimental Information	Dosage/Administration Period	Symptoms	Results’ Summary	Reference
*L. rhamnosus* administration in obesity and related metabolic disorders				
Four-week-old male C57BL/6J mice fed with a high-fat diet (HF)	Postbiotics from *L. rhamnosus* HF01 strain were daily administered (0.1 mL/10 g body weight), for 12 weeks.	Obesity symptoms, such as increased body weight, high Lee index, and abdominal fat ratio, lower glucose tolerance, and alterations in the gut metabolic profile	Decreased level of lipopolysaccharides. The colon tissue integrity was preserved. The mRNA levels of NLRP3, Caspase-1, and TLR4 were inhibited. Protein expressions of Occludin, Claudin, and ZO-1 increased. The expression of genes encoding proteins involved in glucose metabolism was restored to normal levels. Liver insulin sensitivity was alleviated by remodeling the IRS/PI3K/Akt pathway.	[88]
8–9-weeks-old Wistar rats	Rats fed for 6 weeks with high-fat high-fructose enriched with *L*GG or heat-inactivated *L*GG (postbiotics) (10^9^ CFU/day).	Body fat deposition. Increase the liver size. Increase serum levels of hepatic transaminases. Lower concentration of species in the gut.	Firmicutes and Verrucomicrobia were the predominant taxa. Bacteroidetes were not found. Interaction between *L. rhamnosus* and *Blautia glucerasea* favorize the reduction of TG and TyG index, Hepatic steatosis was reduced. Inflammation in the liver decreased. Visceral fat deposition decreased.	[91]
*L. rhamnosus* administration in major depressive disorders (MDD)				
Chronic ethanol exposure (CEE) mouse model Male C57BL/6 mice (3-month-old); 12 weeks of ethanol exposure	Starting with week 11, mice received daily gavage of *L*GG (1 × 10^10^ CFU/mL, 10 μL/g) for three consecutive weeks. Then, for another three weeks, they received a cocktail of antibiotic (ampicillin, neomycin, metronidazole and vancomycin; 10 µL/g).	Anxiety, depression-like behavior with cognitive impairment	Antibiotic treatment reduced inflammation and behavioral symptoms in CEE mice. The gut microbiota in CEE mice contributed to inflammation and behavioral dysfunction. *L*GG treatment decreased inflammatory cytokine levels (TNF-α, IL-1β, IL-6) in both the peripheral systems and specific brain regions (striatum, amygdala, prefrontal cortex, hippocampus). *L*GG highlighted anti-inflammatory effects and neuroprotective potential.	[98]
200 g of Wistar Albino male rats, 2-month-old. Chronic unpredictable mild stress (CUMS) protocol was applied for 8 weeks (such as, 45° cage tilt, wet sawdust for 21 h, leaving alone in dark cage for 1–2 h, immobilization at 4 °C for 3 h, food and water deprivation for 24 h, restricted access to food for 1 h, etc.) Also, moderate levels of anxiety were induced using dim light, and unfamiliar places.	Gavage with *L*GG in a concentration of 15 × 10^8^ CFU/mL/day for 21 days. The effects of *L*GG administration were compared with those of bupropion (20 mg/kg/day) and venlafaxine (20 mg/kg/day).	Tendency to avoid luminous places and to discover new areas	LGG reduced weight loss during stress. Brain-derived neurotrophic factor level in the hippocampus significantly increased in the rat group treated with *L*GG compared with the control or with the stressed individuals, where its level decreased. Treatment with *L*GG and venlafaxine increased the gene expression of DRD1, enhanced the expression of both adrenergic receptor alpha-2A (ADRA2A) and serotonin receptor 5-HT1A. *L*GG was the only treatment effective in increasing the time of social interaction and locomotor activities in the stress-induced group.	[99]
Parkinsons’ disease				
Healthy male C57BL/6 mice of 8- to 10-week-old	10^8^ CFU/mL of *L. rhamnosus* E9 strain (isolated from healthy infant feces) by oral gavage for 15 days. For 5 consecutive days intraperitoneal injection of 30 mg/kg MPTP-HCl were administrated.	Motor dysfunction, dopaminergic damage in the mouse brain, chronic inflammatory state	MPTP injections affected the locomotor activity and exploration behavior even in the probiotic group. After, the E9 strain administration the muscle strength was preserved, and cataleptic symptoms were reduced. MPTP administration significantly decreased the tyrosine hydrolase expression and the dopamine level in the striatal tissue. The expression of dopamine transporter (DAT) also decreased in MPTP-treated mice, while D1-like receptor (DR1) expression increased. The E9 strain administration preserved DR1 and DAT expressions to the control levels, suggesting protective or stabilizing effects on dopaminergic signaling pathways. E9 may exert neuroprotective effects by attenuating the oxidative damages which were observed after MPTP administration.	[101]
Effects on the cerebrospinal fluid (CSF)				
Adult male Sprague Dawley^®^ rats (250–265 g)	*L*GG cells concentrated in reverse osmosis water to 3.34 × 10^7^ CFU/mL and administrated daily for 21 days. The goal was to administrate approximately 1.17 × 10^9^ CFU of *L*GG per animal.	Healthy individuals	The levels of the extracellular matrix proteins from CSF (cochlin, NPTXR, reelin, Sez6l, and VPS13C) increased, while the levels of CPQ, and IGFBP-7 decreased. The relative expression of IL10 mRNA in the hippocampus also increased. The *L*GG administration reshapes the extracellular matrix proteins in order to support synaptic plasticity, influencing glutamatergic signaling pathway and promoting anti-inflammatory state within the CNS.	[102]

Treatment with *L. rhamnosus* E9 strain maintained DR1, and dopamine transporter (DAT) expression at levels comparable with those of the healthy individuals, indicating a neuroprotective stabilization of dopaminergic pathways. Additionally, the MPTP administration determined elevated reactive oxygen species (ROS) levels, indicating major oxidative damages to proteins, membranes, and DNA, and α-synuclein misfolding in the brain, effects that were significantly attenuated after E9 strain administration [101,103]. These findings suggest that *L. rhamnosus* E9 administration alleviates oxidative stress and preserves dopaminergic function, highlighting its potential as therapeutic agent for PD.

Recent studies have shown that the extracellular vesicle of *Lactobacillus* spp., as part of their normal metabolic functioning, serve as mediators in cell–cell communication networks, having the capacity of crossing different biological barriers (blood, brain) and interacting directly with the neurons and glia cells [102]. In this context, Loupy et al. [102] highlighted that, after 21 days of *L*GG delivery to rats via drinking water, the levels of five proteins (cochlin, NPTXR, reelin, SEZ6L, and VPS13C) from the cerebrospinal fluids (CSF) increased while the abundance of CPQ, and IGFBP-7 decreased [102,104] (Table 1, Figure 2). These proteins are linked to synaptic plasticity and glutamatergic signaling and, together with the *L*GG immunoregulatory effects, may contribute to the development of a stress-resilient neural phenotype [105]. In addition, the *L*GG administration may modulate the main key pathways that are disrupted in Alzheimer’s disease (AD) [106]. Given the overlap with the MDD, these findings underscore the *L*GG strain potential as a therapeutic strategy targeting the common mechanisms shared in both disorders, MDD and AD.

## 4. Strategies and Systems for Efficient *L. rhamnosus* and Its Metabiotics Delivery for Functional Purposes

Functional foods are those that, beyond their nutritional properties, have been promoted as important providers of health benefits after consumption. Food products containing probiotics constitute a major category among functional foods, for which, due to the increasing interest in a healthy lifestyle and a high prevalence of gastrointestinal disorders, consumer demand is rapidly growing [107]. The main challenge in the development of such food products is to ensure sufficient levels of probiotic microorganisms at the end of the production process and during storage, so that they can exert the expected beneficial effects on consumers after consumption. To reach the human intestine in a concentration which exerts the probiotic effect, daily intake of probiotic microorganisms is generally recommended to be between 10^8^ and 10^9^ CFU, which means that the delivering food products should contain at least 10^6^ CFU per mL or g [108].

Furthermore, in the manuscript of Guarner et al. [109], comprehensive information about the commonly used probiotic products, their constituent strains, and recommended daily intake dosage is also provided. More precisely, consumption of 50 g of probiotic cheese containing the *L*GG strain has been documented to treat oral candidiasis, or a dosage of 10^10^ CFU once per day obtained from a multi-strain probiotic product containing *L*GG, *L. rhamnosus* LC705, *Propionibacterium freudenreichii* ssp. *shermanii* JS DSM7067, and *Bifidobacterium animalis* ssp. *lactis* Bb12 DSM 15954 is recommended to improve irritable bowel syndrome symptoms, respectively.

Formulations combining live probiotics and selected prebiotics are advanced preparations defined as synbiotics which offer enhanced health benefits beyond what probiotics can achieve alone [110]. The new concept of tribiotics refers to a combined strategy that integrates the synbiotics and postbiotics into a single formula aiming to address synergistic effects on gut microbiome modulation, and therefore health benefits on the host [111]. Currently, the encapsulation of synbiotics is an emerging strategy which improves stability, viability, and targeted delivery of the two enclosed components.

*L. rhamnosus* has the capacity to adapt to different food matrices and to synthesize important metabolic compounds with impact on food functionality. Dairy products are, perhaps, the traditional vehicle of delivering probiotics to consumers. Fermented yogurt supplemented or not with prebiotics (yeast extract alone or in combination with inulin) and maintained under refrigeration conditions (4 °C) for 1 month, ensured a viability of *L. rhamnosus* GR-1 strain at a minimum of 1 × 10^7^ CFU/mL [112]. Other studies aimed at extending the means of *L. rhamnosus* delivery to consumers have been focusing on obtaining functional cereal-based products. Buckwheat mashes proved to be a good substrate for facilitating LGG growth, even in co-culturing conditions with other LABs. The functional food was able to maintain a probiotic cells level higher than the minimum required throughout fermentation and storage [113]. Fermentation for 21 days of rice pudding supplemented with prebiotics, such as short and long chain inulin, or oat, resulted in a level of *L. rhamnosus* GR-1 cells higher than 1 × 10^8^ CFU/mL. The addition of prebiotics, especially short chain inulin (4% *w*/*w*), increased the hedonic score of the newly developed functional product, improving its sensorial properties, such as flavor, sweetness, and texture, and overall acceptability [114]. Production of pasta and fresh noodles fortified with *L*GG resulted in levels of the probiotic bacteria of 7.5 log CFU/g, and 8.92 log CFU/g, respectively. Additionally, noodles boiling maintained the probiotic efficacy of the product, the concentration of *L*GG being reduced to 6.88 log CFU/g after 7 min of thermal treatment [115].

To limit the negative impact of certain stress factors encountered in the food matrices (fluctuating temperature, pH, relative humidity, or oxygen level) and the digestive tracts of consumers (electrolytes, gastric acid, bile salts, digestive enzymes, or antimicrobial peptides) on the level of the probiotic cells [116], different encapsulation systems have been explored (Table 2). Choosing the optimum delivery solution depends on several factors; the most important ones are the method of encapsulation, the afferent processing conditions, and the wall material composition. In turn, the encapsulation method has to be chosen according to different factors, such as capsules’ size, microparticles’ biocompatibility, and intention to be applied at a larger scale. Nonetheless, the encapsulation material must be suitable for the encapsulation technique and exhibit good film-forming ability, solubility, stability, digestibility, and releasing properties, as well as suitability for human consumption [5,117].

Among the currently available encapsulation techniques are spray drying, freeze-drying, emulsification, coacervation, liposomal delivery, and electrospraying [5].

The efficiency of the encapsulation methods applied to protect *L. rhamnosus* strain against the adverse conditions was reported to vary in terms of process parameters, percentage of cells entrapment, or physical properties of the capsules (moisture content, size, releasing efficiency). While freeze-drying led to a higher encapsulation percentage, electrospraying resulted in more compact and uniform microparticles, which ensured better protection of *L*GG against the acidity and cold temperature conditions related to yogurt storage. Moreover, the yogurt functionalized with the microcapsules obtained through electrospraying showed better sensory attributes, especially texture and taste, and higher consumer acceptability compared to the same product containing free *L. rhamnosus* cells or encapsulated ones by freeze-drying [125].

Co-encapsulation of *L. rhamnosus* with bioactive compounds as a way to simultaneously provide multiple health benefits to consumers, as well as improve the probiotic cells survivability when exposed to harsh conditions, has been also addressed. Freitas de Lima et al. [123] developed biphasic way based microparticles by spray drying *L*GG with β-carotene encapsulated in liposomes. The multifunctional capsules, which retained satisfactory levels of β-carotene (64.48%) and a high level of viable probiotic bacteria (>10^9^ CFU/g) and had adequate physicochemical properties, exhibited great stability at room temperature for 90 days and important anti-inflammatory effects in Wistar rats with induced paw edema and pleurisy [123].

Co-encapsulation of probiotics with polyphenols is a promising strategy to maximize the potential of each of the microcapsules’ components. Polyphenols can protect probiotics against the unfavorable conditions met during digestion, while probiotics can contribute to the increase of the stability and bioavailability of the prebiotics [126]. Further, Camelo-Silva et al. [127] co-encapsulated *L. rhamnosus* with extract of beet residue (EB) for use in fortifying white chocolate. Although the multifunctional capsules showed a higher reduction in probiotics’ viability after being exposed to the simulated mouth, stomach, and ileum conditions compared to the free and encapsulated without EB ones, due to a softer texture of the chocolate, in the colon environment, after 48 h of fermentation, the cells level exceeded 11 log_10_ CFU/g. This spectacular proliferation of *L. rhamnosus* cells might be attributed to the prebiotic effect of EB, as an important fraction of the phenols is not absorbed in the small intestine and can reach the colon, where it becomes a valuable substrate for the resident microbiota [127].

Another approach to deliver *L. rhamnosus* simultaneously with other functional compounds is represented by the absorption of the bovine lactoferrin on the surface of the sodium alginate wall. This system was strengthened with calcium chloride and mineralized with disodium hydrogen phosphate proving an efficiency of ~95% due to the high porosity of the microcapsules and the availability of certain functional groups. The resulting capsules exhibited greater stability during simulated gastric digestion, demonstrating a lower swelling behavior than those made of sodium alginate alone or sodium alginate and calcium chloride and preserving better the viability of *L. rhamnosus* cells [128].

Combining the conventional wall materials with prebiotics at different proportions can increase the survivability of *L. rhamnosus* cells during the encapsulation process, storage, or passage through the upper gastrointestinal tract. Other used matrices for *L*GG encapsulation were represented by the whey protein isolate (WPI) combined with fructooligosaccharides (FOS) and galactooligosaccharides (GOS) [129], or inulin embedded in double emulsions (W/O/W), who can contribute to an extended preservation of the subcellular structures [130]. It was postulated that, under acidic conditions, inulin can form insoluble aggregates within the composite network and cover its pores, preventing the diffusion of the H^+^ ions within the core of the microcapsules [122].

Exclusive use of plant derived wall materials as alternatives to the frequently employed encapsulation agents, such as whey proteins or gelatin, has also led to the obtaining of promising *L. rhamnosus* delivery solutions. Santos et al. [124] obtained various wall material formulations by using pea protein, pectin, tapioca flour, and maltodextrin. The formulations which ensured the highest encapsulation efficiency and provided the greatest protection to *L*GG cells during storage and in vitro digestion consisted of different combinations and percents of maltodextrin, pea protein, tapioca flour, and pectin [124] or the carob flour with pectin [118] (Table 2).

However, limited information regarding the encapsulation of metabiotic products obtained from *L. rhamnosus* strains fermentation is currently presented in detail in scientific literature. In a study of particular interest, Lu et al. [131] incorporated the *L. rhamnosus* HN001 strain and its postbiotic products resulting from short-term fermentation into a composite film matrix composed of whey protein isolate, gum arabic, isomaltooligosaccharides, and glycerol. Their results emphasized significantly enhanced antioxidant and antimicrobial activities, along with maintaining the viability of the probiotic strain during storage at 4 °C.

When compared to each other, the encapsulation methods may present several advantages and disadvantages in terms of process duration, equipment complexity, costs, suitability for the materials to be encapsulated, and resulting capsules’ properties. Some advantages and disadvantages of the mainly employed techniques in probiotics encapsulation are illustrated in Table 3.

These findings offer new research directions for the incorporation of metabiotic products into edible films, as well as encapsulation, aiming to enhance not only the probiotics viability, but also the shelf life of foods.

## 5. Challenges and Limitations

Despite the researchers’ interest and effort in creating new genetically modified strains of *L. rhamnosus*, many limitations still block their use in the food industry sector. First of all, the literature has reported a limited number of recombinant *L. rhamnosus* strains due to the lack of efficient CRISPR-Cas systems, or the instability of plasmids after integration in the probiotic genome without maintaining selective pressure. On the other hand, even when the genetic modification was successful, no further research showing how these strains behave under human physiological conditions was made, including horizontal gene transfer risks, interactions with the host microbiota, or host immunity, due to the complexity of these types of experiments and the safety, regulatory, and ethical aspects. Moreover, in the process of food manufacturing, some important aspects regarding high lactose or glucose concentrations, the interaction with complex food matrices (proteins, fats, fibers), or storage under refrigeration conditions may compromise the GMO stability or minimize the fermentation rate, altering the organoleptic characteristics, which can further generate unpredictability with respect to product quality and consumer acceptance. Of course, these approaches are also necessary even for the unmodified strains when encapsulated for human consumption, to offer functional benefits. Many of the limitations about the releasing aspects of encapsulated probiotics were prior presented by [138], but additional challenges remain, particularly for *L. rhamnosus* strains. Precisely, long-term human studies may respond to the uncertainties regarding the colonization of encapsulated *L. rhamnosus* after prolonged consumption, or whether, following capsule degradation, these strains are able to remain stable under the microbiome inter-individual variability. Finally, to ensure the therapeutic effect, it is better to begin the intervention by determining the host’s exact microbiome composition, which can be assessed through next-generation sequencing (NGS), and then to stabilize an efficient and personalized probiotic/synbiotic formulation to enhance the health benefits.

## 6. Conclusions and Future Perspectives

By applying modern techniques like metagenomic sequencing, it is possible to select specific *L. rhamnosus* strains or probiotics consortia to use them in personalized formulations adapted to the specific needs of each person, considering their health profile. Beyond this, the use of genetic engineering, especially CRISPR-Cas technologies, to create new *L. rhamnosus* strains with enhanced traits has a high potential to revolutionize the food industry, contributing to the development of next-generation of functional foods.

These innovative gene editing technologies could also be able to design specific *L. rhamnosus* strains to target specific human diseases, including cancer, inflammatory disease, or neurodegenerative disorders [139,140].

On the other hand, the probiotic *L. rhamnosus* strains’ metabolites, as alternative promoters of health and wellbeing, require a deeper understanding of the underlying molecular mechanisms in order to prove that their safety and efficacy are comparable to those of the viable counterparts. Further, selection and metabolic activity coordination of *L. rhamnosus* strains for the synthesis of bioactive compounds with clinical implications, particularly on the gut–brain axis as new psychobiotic alternatives, are critical aspects to be considered in personalized health interventions.

Moreover, integrating synthetic biology approaches with encapsulation strategies will allow specific delivery of metabolites from micro- or nano-structured materials, enabling functional advantages for food products, such as improved safety, extended shelf life, or enhanced sensory and nutritional profiles. Furthermore, by combining prodrug nanomicelles with *LGG*, it was possible to improve probiotic survival and therapeutic efficacy in ulcerative colitis [141]. This approach opens a new direction focused on developing specialized nanocarriers for engineered probiotics to enable targeted therapies.

The bioactive compounds co-encapsulated with *L. rhamnosus* may modulate not only the gut microbiota composition and the immune responses, but also the interaction with the host metabolic pathways responsible for energy regulation, lipid metabolism, and inflammatory processes. Ultimately, future research should also focus on consumer perception, ethical considerations, and risk assessments under existing regulatory frameworks.

## Figures and Tables

**Table 2:** Encapsulation methods to enhance *L. rhamnosus* functionalities across gastrointestinal.

*L. rhamnosus* Concentration and the Encapsulating Core Material	Wall Material	Encapsulation Technique (Parameters)	Encapsulation Efficiency	Functionalities	Reference
9.85 log CFU/g *L. rhamnosus* 1.0320 strain + 12% polyvinyl alcohol solution: 11% pectin solution (9:1)	14% Eudragin S100 in anhydrous ethanol: N,N-dimethylacetamide solution (9:1)	Coaxial electrospinning (syringes connected to 16-gauge needles with inner and outer diameters of 0.35 mm and 1 mm, respectively Flow rates of shell and core solutions of 1.6 mL/h and 0.4 mL/h, respectively Voltage of 15 kV; distance between the coaxial needle and collector plate of 14 cm)	91.65 ± 0.92%	Increase in probiotics’ survivability after exposure to simulated gastric (pH 3), intestinal (pH 6.8), and continuous gastrointestinal fluids with 11.15%, 12.50%, and 24.30%, respectively.	[100]
6–7 log CFU /mL *L*GG	2.93 g carob flour + 50 mL 0.5% NaCl solution + 2.5 mL 1% pectin solution + 10 mL 0.5% BaCl_2_ solution	Emulsion method (temperature at 26.97 °C; time: 33.24 min)	79.51 ± 0.36%	Increase in the viability of the *L. rhamnosus* cells after exposure to gastric (pH 2.0, 2 h), intestinal (pH 7.0, 2 h), and sequential digestion conditions by 56.74%, 5.35%, and 60.93%, respectively. Ensuring good stability of *L. rhamnosus* cells during storage at −24 °C (106.57%) and 4 °C (96.87%) for 28 days.	[118]
10.62 log CFU /mL *L. rhamnosus*	2% (*w*/*v*) starch solution + 0.5% (*w*/*v*) sodium alginate solution injected dropwise on 0.2 M CaCl_2_ solution	Extrusion (hardening time: 20 min)	85 ± 3%	Probiotics’ survivability of 82.97% and 84.21% after 2.5 h of exposure to simulated gastric juice (pH 2) and intestinal fluid (pH 6.8), respectively.	[119]
9 log CFU /mL *L. rhamnosus* ATCC 7469 strain	3.5% (*w*/*v*) sodium alginate coated with 0.7% (*w*/*v*) chitosan	Extrusion in 2% CaCl_2_ solution (10 mL syringe; gelation time: 30 min) followed by vacuum freeze-drying in the presence of cryoprotectants (15.7% (*w*/*v*) skimmed milk powder, 11.1% (*w*/*v*) trehalose, 9.1% (*w*/*v*) glycerol, and 3.5% (*w*/*v*) sodium glutamate)	93.9%	Increase in the probiotic cells’ survivability with 38.62%, after exposure to simulated oral cavity (pH 7.0), stomach (pH 2.0, 2 h), and intestinal (pH 7.0, 3 h) conditions, respectively. Probiotic cells release rate of 93.13% after incubation in simulated colonic fluid for 150 min. Increase in relative cell viability after storage for 60 days at 4 °C and 25 °C by 0.16 and 0.17, respectively.	[120]
9 log CFU /mL *L*GG	2% (*w*/*v*) chitosan solution (CHI)/0.2% (*w*/*v*) zein/tween-80/fucoidan nanoparticles dispersion (ZTFD) (a four-layer encapsulation—(CHI/ZTFD)_2_-LGGm	Layer-by-layer encapsulation	-	Increase in probiotic cells’ survivability after freeze-draying by 18.44%, and thermal treatment at 60 °C for 30 min by 14.33%, respectively. Increase in probiotic cells’ viability after 4 weeks storage at 25 °C, 4 °C, and −20 °C by 36.1%, 6.38%, and 32.21%, respectively. Increase in probiotic cells’ survivability by 3.9% after sequential exposure to gastric (pH 2.0, 120 min) and intestinal (120 min) simulated conditions. A 63.7-fold increase in survivability after in vivo exposure to gastrointestinal conditions.	[121]
*L*GG ± inulin at a concentration of 0.5% *w*/*v* (A/P/I-1), 1% *w*/*v* (A/P/I-2), 1.5% *w*/*v* (A/P/I-3), or 2% *w*/*v* (A /P/I-4)	2% (*w*/*v*) alginate: pectin (9:1, *w*/*w*) + 0.15% (*w*/*v*) CaCO_3_	High efficiency vibration technology (mixture sprayed in 1% CaCl_2_ solution (*w*/*v*) with 300 µm nozzle and filtered using 120 mesh sieve), followed by freeze-drying (48 h)	9.1 ± 0.03 log_10_ CFU/g *L*GG (for A/P) 9.28 ± 0.05 log_10_ CFU/g *L*GG + 2.6% inulin (for A/P/I-1) 9.86 ± 0.08 log_10_ CFU/g *L*GG + 3.4% inulin (for A/P/I-2) 10.09 ± 0.03 log_10_ CFU/g *LGG* + 8.1% inulin (for A/P/I-3) 9.83 ± 0.01 log_10_ CFU/g *LGG* + 5.9% inulin (for A/P/I-4)	Heating at 63 °C for 30 min, and 72 °C for 3 min led to free cells reduction to 3.27 log_10_ CFU/mL, and 3.10 log_10_ CFU/mL, respectively. The survivability of the encapsulated cells after thermal treatments ranged between 3.43 and 5.58 log_10_ CFU/g, and 4.82 and 7.85 log_10_ CFU/g, respectively. Co-encapsulation with inulin led to a lower loss of *L*GG cells viability after storage at 4 °C for 60 days compared to the free cells (~ 2 log_10_ CFU/mL) and the encapsulated ones without inulin (0.62 log_10_ CFU/g). Survivability of *L*GG exposed to simulated gastric digestion (pH 2, 120 min) increased with the inulin content. Compared to the free cells, which were reduced to 3.7 log_10_ CFU/mL, the encapsulated ones showed a higher resistance, being reduced to 6.12–8.11 log_10_ CFU/g.	[122]
*L*GG and β-carotene loaded large multivesicular liposomes (LMVL_B_)	Cheese whey powder	Spray-drying (Inlet temperature: 130 °C and 170 °C; Outlet temperature: 75 °C; Atomization nozzle diameter: 0.7 mm; compressed air flow: 600 L/h)	Biphasic microparticles dried at 130 °C (BDM 130) showed an efficiency of 56.92% LMVL_B_ + 98.50% LGG The BDM 170 highlighted an efficiency of 64.48% LMVL_B_ + 92.99% *LGG*	BDM were highly stable throughout 90 days storage at 25 °C. Administration of 2000 mg/kg of BDM to rats with carrageenan-induced paw edema and pleurisy resulted in a significant inflammation reduction.	[123]
10 log CFU /g *LGG*	5% maltodextrin, 5% pea protein, 5% tapioca flour, and 1% pectin (F1) 10% maltodextrin, 4% pea protein, and 2% tapioca flour (F2) 10% maltodextrin, 2% pea protein, and 4% tapioca flour (F3) 7.5% pea protein, 7.5% tapioca flour, and 1% pectin (F4).	Spray-drying (Two-fluid nozzle with inner diameter of 1 mm; Inlet temperature: 130 °C; Outlet temperature: 57 °C; Feed flow rate: 0.25 L/h; Drying air flow rate: 3 m^3^/min)	Efficiency of 93.60 ± 0.40% for F1, 94.58 ± 0.86% for F2, 90.81 ± 0.56% for F3 and 88.76 ± 0.90% for F4, respectively	All wall material formulation ensured an increase in the probiotic cells survival after treatment at 80 °C for 5 min by at least 4.04%, with F1 achieving the highest survival rate of 65.95%. F2 showed the smallest viability reduction of *L*GG cells of all formulations when exposed to gastric (pH 2.5, 2 h) and intestinal fluids (pH 8.0, 4 h) simulated conditions. F1 formulation ensured the highest cell viability after 7 weeks of storage at 8 °C.	[124]

**Table 3:** Advantages and disadvantages of current encapsulation techniques with relevance for industrial and therapeutic applications.

Encapsulation Technique	Advantages	Disadvantages	References
Spray drying	Instant drying with low degradation effect on the materials to be encapsulated Suitability for various materials (solutions, suspensions, gels, gels, pastes, etc.) Precise control over particles’ size, shape, crystallinity degree, bulk density Simple scale up Lower processing costs compared to freeze-drying	Quite sophisticated design and control mechanisms of the large capacity industrial spray-dryers Difficulty in recovering the dry powders under certain circumstances (e.g., wall deposition) Highly qualified personnel for the equipment operation and maintenance	[132]
Freeze-drying	Low contamination rate Preservation of the oxidable compounds Extended shelf life of the powders due to the water elimination in high proportions (95 to 99.5%) Minimal loss of flavor and nutritional compounds Powders’ storage under atmospheric conditions Easily reconstitutable particles due to their porous structure	Time-consuming process High capital and equipment maintenance costs Low thermal efficiency Difficult modification of the process parameters	[132]
Emulsification	Mild processing conditions suitable for sensitive materials, such as probiotics Low shear stress on probiotics Easy to scale up Simple technique with increased flexibility Low energy requirement	Obtaining particles with non-uniform sizes and shapes Unsuitable for mass production	[133,134]
Coacervation	Obtaining particles with increased stability in low pH food products Controlled release of probiotics in neutral pH environments, such as human large intestine, due to the particles’ instability in these conditions	Probiotics encapsulated through complex coacervation generally require a subsequent drying step to be more easily handled, which may affect their viability and imply high production costs	[135,136]
Liposomal delivery	Control of the particles’ size Good physical stability Easy preparation of liposomes Controllable ingredients’ release Obtaining uniform pellets at an industrial level Easy to scale up Cost-effective	Thermodynamic instability of the particles Possibility of structure disruption during food processing and release of the entrapped materials	[134]
Electrospraying	Obtaining particles with uniform and compact morphology and increased resistance to acidic environments Operation under mild conditions (e.g., ambient temperature), which helps protect the probiotics’ viability User-friendly technology Scale up potential	Limited variants of encapsulation materials suitable for human consumption Insufficient research on the release mechanism of the encapsulated material under human gut conditions	[137]

## Data Availability

No new data were created or analyzed in this study. Data sharing is not applicable to this article.

## Abbreviations

The following abbreviations are used in this manuscript:

ACC	Acetyl-CoA Carboxylase
ALE	Adaptive Laboratory Evolution
ADRA2A	Adrenergic Receptor Alpha-2A
A/P/I	Alginate/Pectin/Inulin
AD	Alzheimer’s Disease
ADAM	A Disintegrin and Metalloproteinase
ARGs	Antibiotic Resistance Genes
BA	Bile Acids
BDM	Biphasic Dried Microparticles
BDNF	Brain-Derived Neurotrophic Factor
CEA	Carcinoembryonic Antigen
CPSs	Capsular polysaccharides
CXCL	Chemokine (C-X-C motif) ligand
CHI	Chitosan Solution
CEE	Chronic Ethanol Exposure
CUMS	Chronic Unpredictable Mild Stress
CFS	Cell-Free Supernatant
CSF	Cerebrospinal Fluids
CD8	Cluster of Differentiation 8
CRISPR	Clustered Regularly Interspaced Short Palindromic Repeats
cGAS	Cyclic GMP-AMP synthase
DR	Dopamine Receptor
DAT	Dopamine transporter
EGFR	Epidermal Growth Factor Receptor
FAS	Fatty Acid Synthase
FOS	Fructooligosaccharides
GABA	Gamma Aminobutyric Acid
GE	Gelatin Solution
HFD	High-Fat Diets
HB-EGF	Heparin-Binding Epidermal Growth Factor
ICB	Immune Checkpoint Blockade
IECs	Intestinal Epithelial Cells
IgA	Immunoglobulin A
IGFBP-7	Insulin-like Growth Factor-Binding Protein 7
IRS/PI3K/Akt	Insulin receptor substrate/Phosphoinositide 3-kinase/Protein kinase B
α-KG	α-Ketoglutarat
*L*GG	*Lacticaseibacillus**rhamnosus GG*
LMVL_B_	Large Multivesicular Liposomes
LPS	Lipopolysaccharides
LC-MS	Liquid Chromatography-Mass Spectrometry
LysoPC	Lysophosphatidylcholine
MDD	Major Depressive Disorder
MGEs	Mobile Genetic Elements
MHC II	Major Histocompatibility Complex Class II
MPTP	1-methyl-4-phenyl-1,2,3,6-tetrahydropyridine
MLN	Mesenteric Lymph Nodes
MRE	Multidrug- Resistant *Enterobacteriaceae*
NLs	Nanoliposomes
NPTXR	Neuronal Pentraxin Receptor
NLRP3	Nucleotide-binding domain, Leucine-rich family, Pyrin domain containing 3
PARP	Poly(ADP-ribose) Polymerase
PSD-95	Postsynaptic Density protein-95
PAM	Protospacer Adjacent Motif
ROS	Reactive Oxygen Species
SEZ6L	Seizure 6-Like Protein
5-HT1A receptor	Serotonin 1A receptor
SBS	Short Bowel Syndrome
SCFAs	Short Chain Fatty Acids
SA	Sodium Alginate
SPF	Specific Pathogen-Free
STING	Stimulator of Interferon Genes
SYN	Synaptophysin
TyG	Triglyceride Glucose
TLR	Toll-Like Receptor
TNF	Tumor Necrosis Factor
VPS	Vacuolar Protein Sorting
W/O	Water in Oil Emulsification
ZO-1	Zonula Occludens-1
ZTFD	Zein/Tween-80/Fucoidan nanoparticles Dispersion
